# BCG-Mediated Bladder Cancer Immunotherapy: Identifying Determinants of Treatment Response Using a Calibrated Mathematical Model

**DOI:** 10.1371/journal.pone.0056327

**Published:** 2013-02-25

**Authors:** Cyrill A. Rentsch, Claire Biot, Joël R. Gsponer, Alexander Bachmann, Matthew L. Albert, Romulus Breban

**Affiliations:** 1 Department of Urology, University Hospital of Basel, University of Basel, Basel, Switzerland; 2 Unité d'Immunologie des Cellules Dendritiques, Institut Pasteur, Paris, France; 3 École Nationale Supérieure des Mines de Paris, Paris, France; 4 Institut National de la Santé et de la Recherche Médicale U818, Paris, France; 5 Unité d'Epidémiologie des Maladies Emergentes, Institut Pasteur, Paris, France; West Virginia University, United States of America

## Abstract

Intravesical Bacillus Calmette Guérin (BCG) immunotherapy is considered the standard of care for treatment of non-muscle invasive bladder cancer; however the treatment parameters were established empirically. In order to evaluate potential optimization of clinical parameters of BCG induction therapy, we constructed and queried a new mathematical model. Specifically, we assessed the impact of (1) duration between resection and the first instillation; (2) BCG dose; (3) indwelling time; and (4) treatment interval of induction therapy – using cure rate as the primary endpoint. Based on available clinical and *in vitro* experimental data, we constructed and parameterized a stochastic mathematical model describing the interactions between BCG, the immune system, the bladder mucosa and tumor cells. The primary endpoint of the model was the probability of tumor extinction following BCG induction therapy in patients with high risk for tumor recurrence. We theoretically demonstrate that extending the duration between the resection and the first BCG instillation negatively influences treatment outcome. Simulations of higher BCG doses and longer indwelling times both improved the probability of tumor extinction. A remarkable finding was that an inter-instillation interval two times longer than the seven-day interval used in the current standard of care would substantially improve treatment outcome. We provide insight into relevant clinical questions using a novel mathematical model of BCG immunotherapy. Our model predicts an altered regimen that may decrease side effects of treatment while improving response to therapy.

## Introduction

Adjuvant treatment of non-muscle invasive bladder cancer (NMIBC) using intravesical Bacillus Calmette-Guérin (BCG) after transurethral resection was established empirically almost 40 years ago [1]. While BCG therapy remains the standard of care, critical parameters influencing treatment outcome are still poorly understood. In a previous study, we constructed a mathematical model that was calibrated to available clinical data to evaluate the ability of the innate immune system as the principle effector arm responsible for response to therapy. We demonstrated that the effector function of the innate immune response is not potent enough to yield the cure rates observed in clinical practice [2]. We therefore concluded that components of the adaptive immune system must play a critical role in tumor elimination. Adaptive immune effector cells (e.g., T lymphocytes) are distinct from most innate populates as they are long-lived, possess properties of antigen specificity and immunologic memory, with the possibility of interacting with multiple target cells during a single round of activation. Estimates based on experimental data suggest that a single cytolytic T lymphocyte has the potential to kill ten target cells before it requires re-activation by an antigen presenting cell [3]. Moreover, the existence of a memory pool of antigen-specific T cells enables a more robust adaptive immune effector cell response upon secondary re-exposure to BCG.

Using a refined mathematical model that includes the adaptive effector functions of the immune system, we address several clinical parameters in order to learn about their impact for an optimal protocol of successful BCG immunotherapy. Specifically, we integrated available clinical and *in vitro* experimental data to construct and parameterize a new stochastic mathematical model describing the interactions between BCG, the immune system, and tumor cells with the primary endpoint being the probability of tumor extinction. We did not aim for precise quantitative results but rather for a robust qualitative understanding that would remain valid for future models that could integrate increasing levels of detail. Herein, we assessed the impact of: (1) varying the time from resection to BCG instillation, (2) modulating the BCG dose used during intravesical instillation, (3) the indwelling time of BCG, and (4) the inter-instillation interval.

Although all parameters influenced treatment outcome, the most surprising result concerned the impact of the inter-instillation interval where simulations suggested that an interval two times longer than the seven-day interval used in the current standard of care, would substantially improve treatment outcome. Our study provides useful insight and testable hypotheses that could lead to improved management in NMIBC.

## Materials and Methods

**Figure 1:**
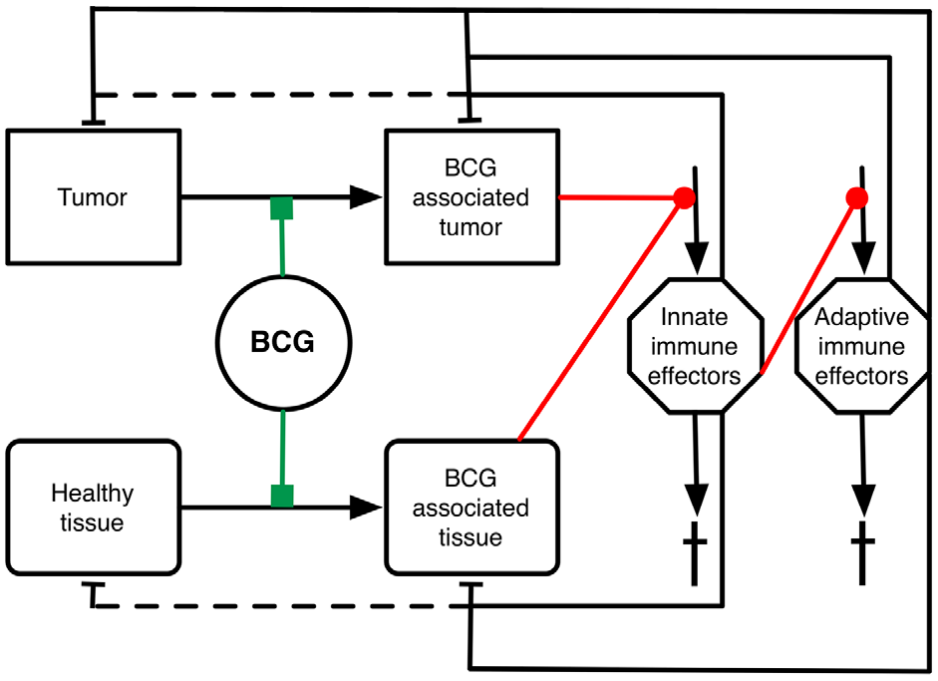
Simplified diagrammatic representation of our mathematical model. The dynamic model represents the interaction between healthy urothelial tissue, tumor cells, and their association with BCG (interaction depicted by green line), which in turn results in the generation of BCG-associated healthy tissue and tumor cells. The latter cell populations possess the capacity to trigger the activation of innate immune effector cells and in turn the priming of adaptive immune effector cells. Red lines ending in circles indicate the input stimuli that influence the recruitment of the indicated immune cell populations. Black arrows indicate either increase (e.g., recruitment or proliferation) or decrease (e.g., death or cell turn-over) of the respective cell population. Lines with blunt ends indicate direct killing of target cell populations (solid lines) or bystander death (dashed lines). The model was parameterized using available clinical and *in vivo* data and tuned to achieve 50% probability of tumor extinction after six weekly intravesical instillations of BCG. A more detailed version of this figure is provided by the **Figure S1** of the supplemental material.

Our mathematical model makes assumptions about the interactions triggered by BCG instillations and simulates the dynamics of populations of cells. Prior to initiation of BCG therapy, three cell populations are present: healthy urothelial tissue, tumor and innate immune cells. The interactions between these cell populations are negligible. The processes that take place for each of these cell populations before therapy are cell migration and/or local proliferation and death (**Figures** **1** and **S1** for schematic; and **Figure S2** for simulation of tumor growth). Of note, activated adaptive immune cells (e.g., antigen-specific T cells) are not present pre-BCG therapy, as their entry requires priming. During BCG instillations, live BCG is capable of actively interacting with cells while dead BCG may be internalized by cells; we refer to BCG as becoming “cell-associated,” to denote either of these two possibilities. As a result of association with BCG, four new cell populations emerge that are linked by dynamic interactions to the previously present cell populations in the bladder: (1) BCG-associated urothelial cells, (2) BCG-associated tumor cells, (3) activated innate immune cells and (4) activated adaptive immune cells. BCG associates to healthy urothelial cells and tumor cells, which in turn triggers increased migration and activation of innate immune cells into the bladder. Innate immune effector cells target BCG associated urothelial and tumor cells, destroying them as well as their neighboring healthy mucosa and uninfected tumor cells [2]. The repeated and boosted inflammation triggers the priming and eventual recruitment of adaptive immune effector cells. Cells of the adaptive immune system may be specific to BCG antigen, thus targeting BCG associated urothelial and tumor cells; but they may also include tumor antigen-specific effector cells, thus permitting the direct targeting of uninfected tumor cells.

The qualitative model presented above was realized as a continuous-time Markov chain where cell populations are given by integer stochastic variables (**Table S1**). Each interaction was defined by its impact on cell populations and assigned a rate of occurrence (**Table S2**). Once the mathematical model was defined, we integrated it numerically using the *efficient tau-leaping algorithm* [4] that we implemented in C/C++. The parameter values of the model (**Table S3**) were established by one of the three methods. First, they were obtained from literature where they were directly estimated from data [2], [5]. Second, they were chosen such that simulations of the cell population dynamics are in agreement with available clinical and *in vivo* data [5]; see **Figure S3** for a sample simulation. Third, they were extracted from calibrating the model such that the probability of tumor elimination is ∼50%, in agreement to clinical data. Hence, for the first time, we obtained a mathematical model that combines immunological and clinical data on BCG immunotherapy. The complete description of the mathematical model and its parameterization are included in the supplemental material.

The model was used for simulating various BCG regimens to assess how tumor extinction is influenced by: (1) varying the time from resection to BCG instillation, (2) modulating the BCG dose used during intravesical instillation, (3) the indwelling time of BCG, and (4) the inter-instillation interval. Each probability of tumor extinction was calculated by running 1,000 stochastic simulations, such that it's resulting 95% confidence interval became reasonably small. The computer code was also employed in uncertainty and sensitivity analyses for assessing the robustness of our results (**Figure S4**).

## Results

**Figure 2:**
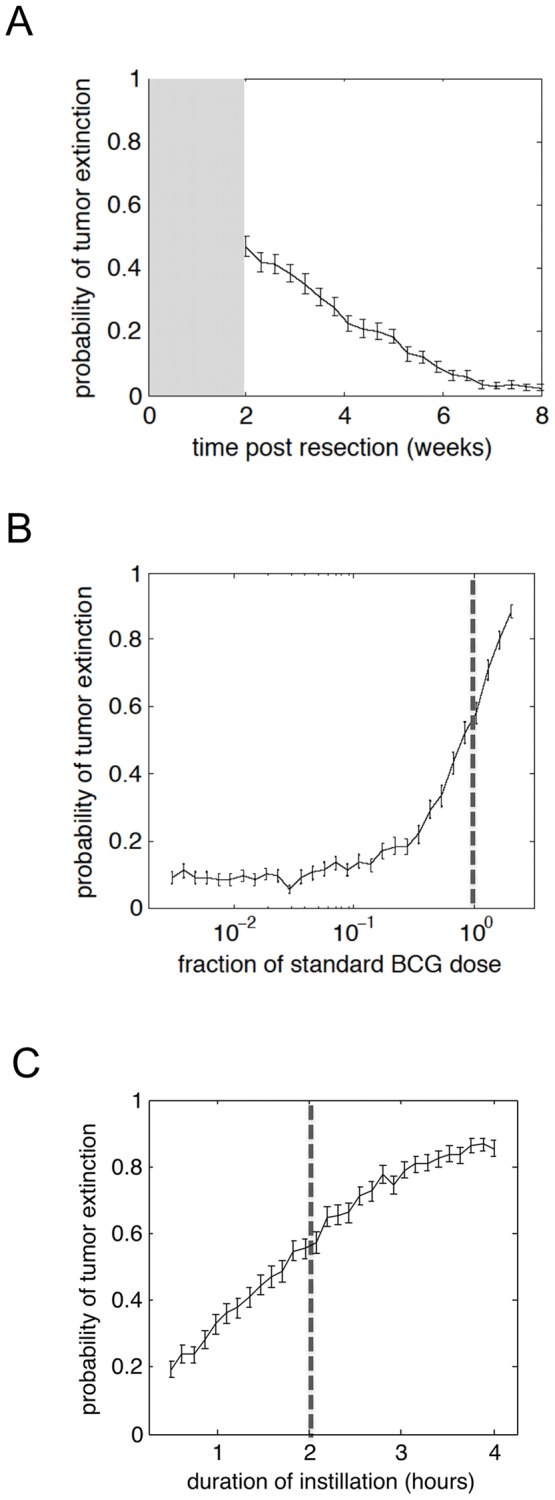
Timing of BCG therapy, BCG dose and dwell time influence probability of tumor extinction. (**A**) Probability of tumor extinction with varying the time from surgery to the start of BCG therapy. The grey shaded area represents the time from surgery to the typical initiation of BCG therapy (i.e., 2 weeks). The cancer growth rate used is based on the clinical observation that high-grade lesions become visible by 3 months in the absence of treatment (detailed definition of tumor dynamics is provided in the supplementary information and **Figure S2**). The probability of tumor extinction after six weekly BCG instillations was modeled as a function of (**B**) BCG dose, and (**C**) BCG dwell time.

Using the parameterized mathematical model, we evaluated treatment parameters believed to influence clinical response. First, we determined the influence of the time interval between surgery and initiation of BCG treatment. The model calculated the probability of tumor extinction after six weekly instillations as a function of the post-resection time (**Figure** **2A**). The shorter the interval from surgery to BCG therapy, the greater is the chance for achieving clinical response.

The model was further queried to determine impact of BCG dose and dwell time in the bladder. We noted a clear positive correlation between BCG dose and probability of tumor extinction (**Figure** **2B**). Strikingly, small changes in the dose of BCG are predicted to have a significant impact on therapeutic success. BCG is commonly delivered at a range between 10^8^ to 10^9^ CFUs/vial. According to the predictive model, such a 10-fold difference in the clinically applied dose might account for ∼40% differential response to treatment. As a corollary, we examined the dwell time (i.e., the duration of BCG instillation). Again, we find that greater exposure of the bladder mucosa to BCG results in an increased probability of tumor extinction (**Figure** **2C**).

**Figure 3:**
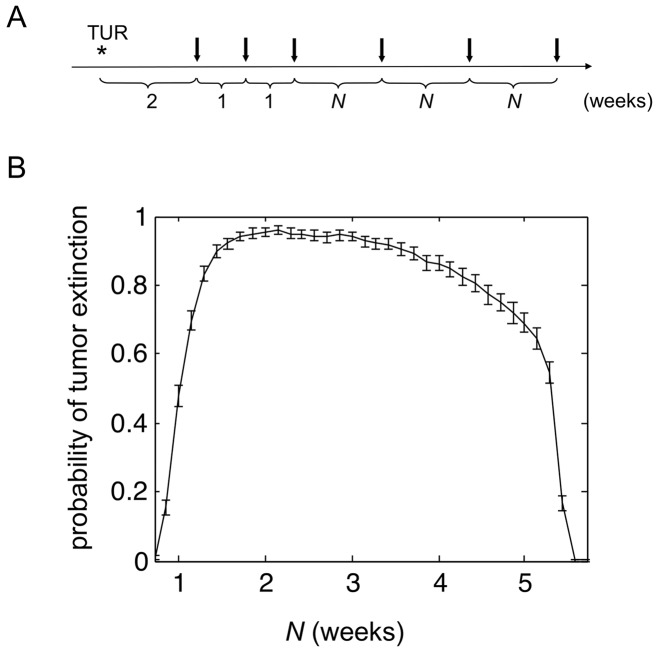
The treatment interval strongly influences the probability of tumor extinction. (**A**) Schematic representation of the modeled surgery time point followed by intravesical treatment time points (arrows). The symbol * indicates the time of transurethral resection (TUR) followed by the 2 weeks interval before BCG therapy starts. Three weekly instillations are given to initiate innate and adaptive immune responses. The time interval between the third through sixth treatment dose was varied and designated by *N* (as measured in weeks). (**B**) Probability of tumor extinction as a function of *N*, the inter-instillation interval during the 3^rd^ – 6^th^ treatment doses. The dotted line marks the outcome after the recommended interval of *N* = 1 week.

Recent data regarding the delayed timing of T cell priming following mycobacterial infection [6], or BCG intravesical instillation [7], suggested that the current treatment schedule may not be properly tuned to the kinetics of adaptive immune responses. Therefore, the impact of varying inter-instillation timing was assessed. We maintained the first three weekly doses, as this is required for priming adaptive immune responses and initiating bladder inflammation [5]; and we modeled the optimal time interval between the third through sixth treatment dose, designated by *N* in **Figure** **3A**. Following from the parameterization conditions, six weekly instillations resulted in the expected cure rate of 50%. Shorter inter-instillation duration negatively impacted clinical response (**Figure** **3B**). Remarkably, the model indicates an optimal treatment interval that is twice longer than the current standard of care, with no negative impact if extended to up to 30 days (**Figure** **3B**).

We performed uncertainty and sensitivity analyses to assess the robustness of our numerical results. We found that the qualitative features of **Figures** **2B & 2C** are preserved with slight variation of parameters. The results presented in the **Figures** **2A** and **3** were also investigated. We found that, upon model recalibration, varying the count of tumor cells at the start of therapy or key model parameters impacts little on our predictions. Details of these analyses are included in the supplemental material (**Figure S4**).

## Discussion

Using a mathematical model we evaluated clinically relevant questions of BCG therapy in NMIBC. The model revealed that therapeutic success depends strongly on the timing of the BCG regimen. An early start of BCG therapy after TUR, combined with an optimal dwell time and treatment inter-instillation interval, do, in fact, have profound influence on treatment outcome, according to our model.

There are no current guidelines advising when to start BCG therapy after surgery. Delay is considered important for the healing of the bladder wall, and the prevention of systemic complications due to BCG therapy. Therefore, most urologists wait 2–6 weeks prior to starting BCG therapy; however, in some studies intravesical treatment has been initiated as early as 1 week after transurethral resection [8], [9]. To date, no prospective comparisons of different delays to start of BCG treatment have been performed. Our model indicates that a prolonged delay in initiating BCG therapy could negatively impact recurrence rates (**Figure** **2A**). Analysis of the model suggested that the increased risk for recurrence was related to outgrowth of residual tumor cells. This result is due to the continued expansion of residual tumor cells post-resection, increasing the burden of disease and challenging the limited kill capacity of the immune system.

Changes in dose and dwell time have been previously discussed in the literature on the basis of reducing side effects. Dose reduction has been assessed in several clinical trials [10], [11], [12], [13], [14], [15]. Reducing BCG dose to one third was considered as a strategy aimed at lowering side effects. This lower dose remained significantly better than mitomycin; whereas one sixth of the BCG dose was not better than the use of mitomycin alone [13], [14]. These findings corroborate our modeling results regarding the influence of dose on the cure rate (**Figure** **2B**). Our model also shows that increasing the dose increases therapeutic success. However, this would probably occur at the cost of enhanced side effects.

Reduction of dwell time has been reported as a possibility for improving therapy and as an alternative to dose reduction in patients with severe side effects [16] but no prospective study has compared BCG dwell time as a variable. Our model indicates that increased dwell time might also influence treatment outcome (**Figure** **2C**). This could be of special importance in patients with minimal symptoms that may benefit from enhanced BCG-mediated immune activation. These results provide a stark reminder of the importance of adhering to, at minimum, the current guidelines for BCG immunotherapy.

Perhaps the most striking finding is the observation that the optimal treatment interval could be twice as long as the current schedule for managing patients (**Figure** **3**). This finding is likely related to the kinetics of T and B cell activation, and the persistence of these relatively long-lived effector cells in the bladder. By extending the treatment interval during the effector phase, we have succeeded in enhancing the time period in which the immune system may exert negative pressure on the residual tumor burden. Only limited information is available from clinical trials where the treatment interval was modified during BCG induction therapy. Studies in mice indicated that the number and timing of the instillations are important in determining different local cytokine profiles, which in turn may influence the qualitative and quantitative recruitment of adaptive effector cells [17]. Such findings in combination with the result of our model support the need for further investigations to determine the optimal timing of BCG instillations. Finally, attention should be paid to the abrupt loss of the treatment effect after a certain interval (**Figure** **3**), a finding that may have implications for the timing of maintenance therapy.

These data are intriguing as the modified treatment regimen engages the afferent immune response during the early phase of treatment and maximally benefits from the long-lived effector potential of the efferent adaptive response. Such a regimen with extended courses of treatment may be better tolerated by patients [18]. Moreover, it is intriguing to consider that extension of the first six doses of BCG could obviate the need for maintenance therapy. Clearly, these findings require validation in pre-clinical experimental models and clinical trials prior to their being adopted for patient management. Nonetheless, we are encouraged by the findings and support the use of mathematical models to establish a framework for optimization of treatment practices.

The field of mathematical modeling in BCG immunotherapy of bladder cancer has emerged only recently [19], [20], [21], [22]. Topics addressed so far have been: BCG dose and number of instillations needed to achieve cure, and the combined effect of IL-2 and BCG. Previous models [19], [20], [21], [22] have a number of limitations, which have been circumvented in this work. First, instead of ordinary differential equations, where the numbers of cells are given by real numbers, we have chosen a stochastic model where the numbers of cells take integer values. Hence, our model is better suited to describe tumor elimination. Second, unlike previous models [19], [20], [21], [22], ours includes the dynamics of BCG-associated healthy urothelial cells, which are more numerous than BCG-associated tumor cells – both serving as initiators of innate and adaptive immunity. Third, we are the first to model the prime boost response of the immune system, a phenomenon believed to be of critical importance for BCG immunotherapy [5]. These methodological advances, in addition to the results presented here, will help advance the use of mathematical models for optimization of immune-based treatment strategies.

## Conclusions

Although by definition imperfect, mathematical models are useful tools for managing empirical knowledge to extract both qualitative and quantitative information. The new insights gained into BCG treatment remain to be clinically tested and validated in order to confirm the coherence of the model and its assumptions. A model like the one presented here, may therefore be considered for the design of new therapeutic strategies and clinical studies.

In sum, our model implicates that a rigorous time management of bladder cancer patients may be crucial for successful BCG treatment and that patients may benefit considerably from an instillation plan with extended treatment intervals. Our results warrant further studies aiming at optimizing BCG therapy in patients with NMIBC.

## Supporting Information

Figure S1 **Flow diagram of the model of interactions between the innate immune system and bladder tumor during BCG instillation.**
(TIFF)

Figure S2 **Simulation of logistic tumor growth, resection and tumor re-growth.** The horizontal dashed line represents the approximate size of the tumor when the tumor is visible on the bladder wall.
(TIFF)

Figure S3 **Simulation of population dynamics of cells during and after a six-week course of intravesical BCG therapy.** BCG (panel **A**), tumor cells (panel **B**), BCG-associated tumor cells (panel **C**), BCG-associated tissue cells (panel **D**), innate effector cells (panel **E**), adaptive effector cells (panel **F**). Note the modeling of the prime/boost response of the innate and adapted immune system occurring after the third instillation (in particular, note panels **E** and **F**).
(TIFF)

Figure S4 **Sensitivity analyses for** **Figs.** **2A** **and** **3** **of the main text.** **A**. Sensitivity analysis of Fig. 3 with changing the rate of loss of adapted effectors, *μ_Ea_*. The blue, black (also shown in Fig. 3) and red curves correspond to *μ_Ea_*  = 0.4, 0.8 and 1.6 day^−1^, respectively. For every parameter set, the model has been re-calibrated such that six weekly instillation of BCG therapy yield ∼50% chance of cure. **B**. Sensitivity analysis of Fig. 3 with varying the number of tumor cells found in the bladder before initiating BCG therapy, *T*(0). The black (also shown in Fig. 3), blue, magenta and red curves correspond to *T*(0)  = 10^6^, 10^5^, 10^4^ and 10^3^ tumor cells. The model has been re-calibrated for each value of *T*(0), accordingly. These numerics suggest that our prediction of improved therapeutic outcome by a two-week inter-instillation interval is robust. **C**. Sensitivity analysis of Fig. 2A with changing the number of tumor cells before initiating BCG therapy, T (0). The black (also shown in Fig. 2A) and red curves correspond to *T* (0)  = 10^6^ and 10^3^ tumor cells, respectively.
(TIFF)

Table S1 **State variables of the model along with their biological description.** The state variables represent counts of various cell types involved in the interactions between the immune system, tumor cells and BCG.
(TIFF)

Table S2 **Stochastic processes and their corresponding rates.**
(TIFF)

Table S3 **Parameters of the model.**
(TIFF)

Text S1 **Detailed description and numerical analysis of the stochastic mathematical model.**
(PDF)
