# Supplementary figures and images for: BCG-Mediated Bladder Cancer Immunotherapy: Identifying Determinants of Treatment Response Using a Calibrated Mathematical Model
Source: PLoS One. 2013 Feb 25;8(2):e56327. doi: 10.1371/journal.pone.0056327 (PMC3581521; doi:10.1371/journal.pone.0056327)

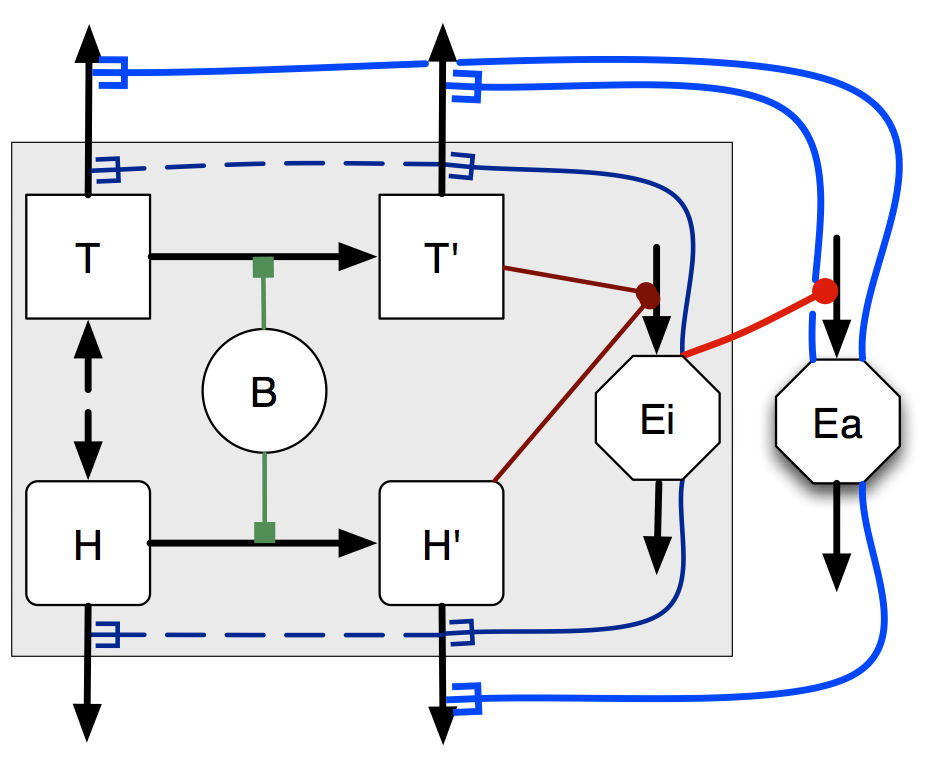

Supplement: Figure S1 — Flow diagram of the model of interactions between the innate immune system and bladder tumor during BCG instillation. (TIFF) [file pone.0056327.s001.tiff]

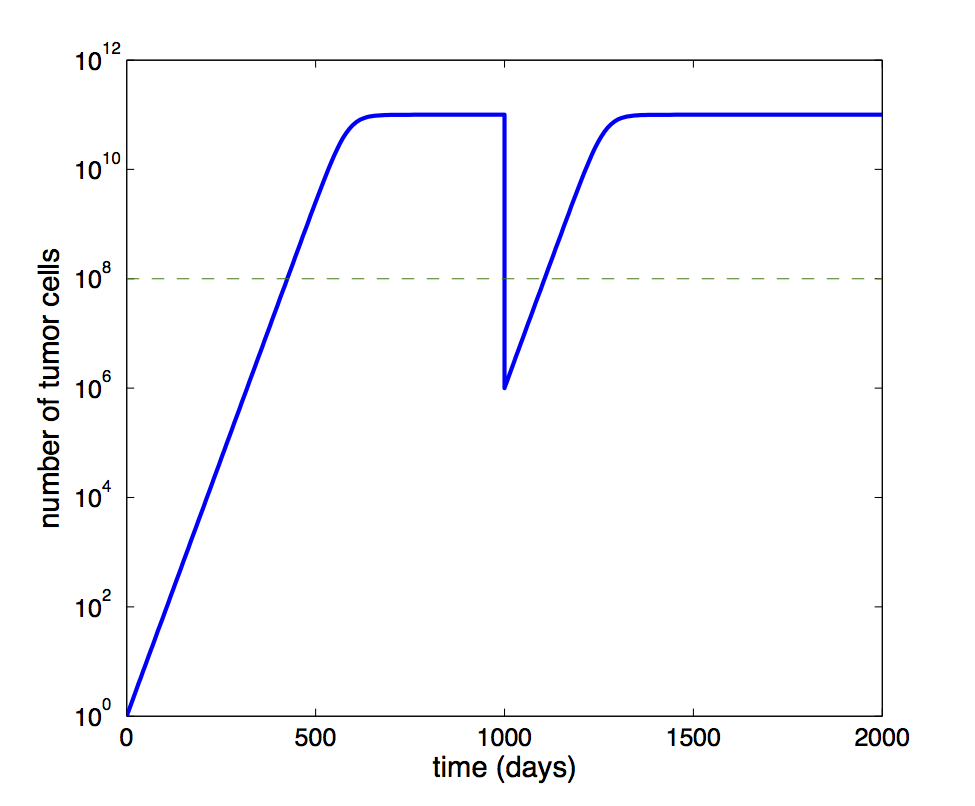

Supplement: Figure S2 — Simulation of logistic tumor growth, resection and tumor re-growth. The horizontal dashed line represents the approximate size of the tumor when the tumor is visible on the bladder wall. (TIFF) [file pone.0056327.s002.tiff]

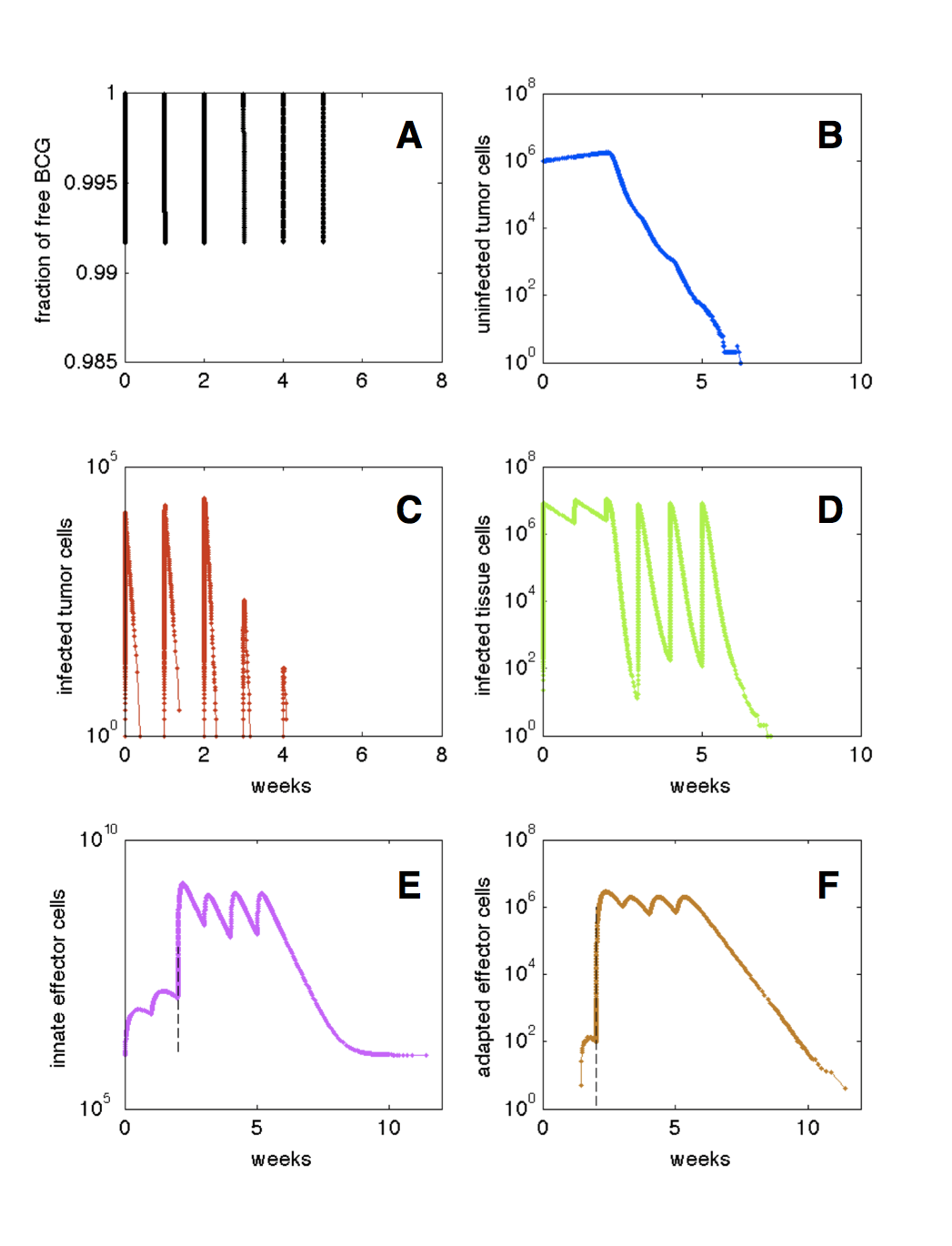

Supplement: Figure S3 — Simulation of population dynamics of cells during and after a six-week course of intravesical BCG therapy. BCG (panel A), tumor cells (panel B), BCG-associated tumor cells (panel C), BCG-associated tissue cells (panel D), innate effector cells (panel E), adaptive effector cells (panel F). Note the modeling of the prime/boost response of the innate and adapted immune system occurring after the third instillation (in particular, note panels E and F). (TIFF) [file pone.0056327.s003.tif]

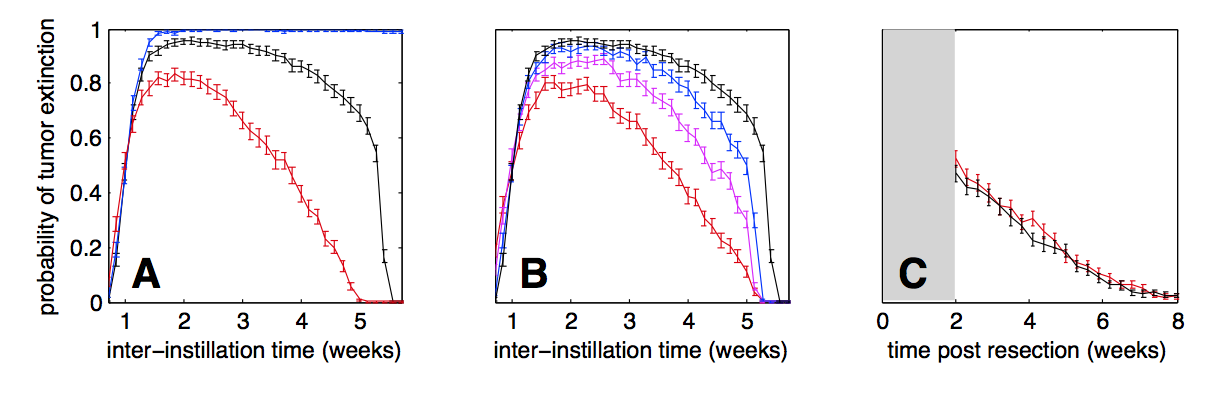

Supplement: Figure S4 — Sensitivity analyses for Figs. 2A and 3 of the main text. A. Sensitivity analysis of Fig. 3 with changing the rate of loss of adapted effectors, μEa. The blue, black (also shown in Fig. 3) and red curves correspond to μEa = 0.4, 0.8 and 1.6 day−1, respectively. For every parameter set, the model has been re-calibrated such that six weekly instillation of BCG therapy yield ∼50% chance of cure. B. Sensitivity analysis of Fig. 3 with varying the number of tumor cells found in the bladder before initiating BCG therapy, T(0). The black (also shown in Fig. 3), blue, magenta and red curves correspond to T(0) = 106, 105, 104 and 103 tumor cells. The model has been re-calibrated for each value of T(0), accordingly. These numerics suggest that our prediction of improved therapeutic outcome by a two-week inter-instillation interval is robust. C. Sensitivity analysis of Fig. 2A with changing the number of tumor cells before initiating BCG therapy, T (0). The black (also shown in Fig. 2A) and red curves correspond to T (0) = 106 and 103 tumor cells, respectively. (TIFF) [file pone.0056327.s004.tiff]

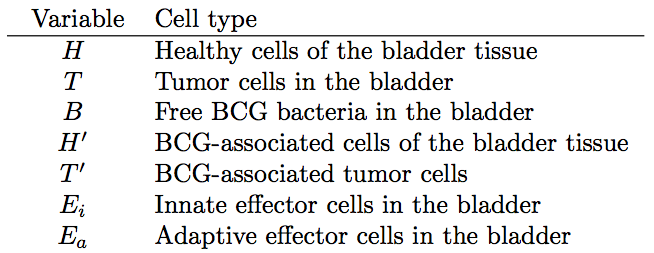

Supplement: Table S1 — State variables of the model along with their biological description. The state variables represent counts of various cell types involved in the interactions between the immune system, tumor cells and BCG. (TIFF) [file pone.0056327.s005.tiff]

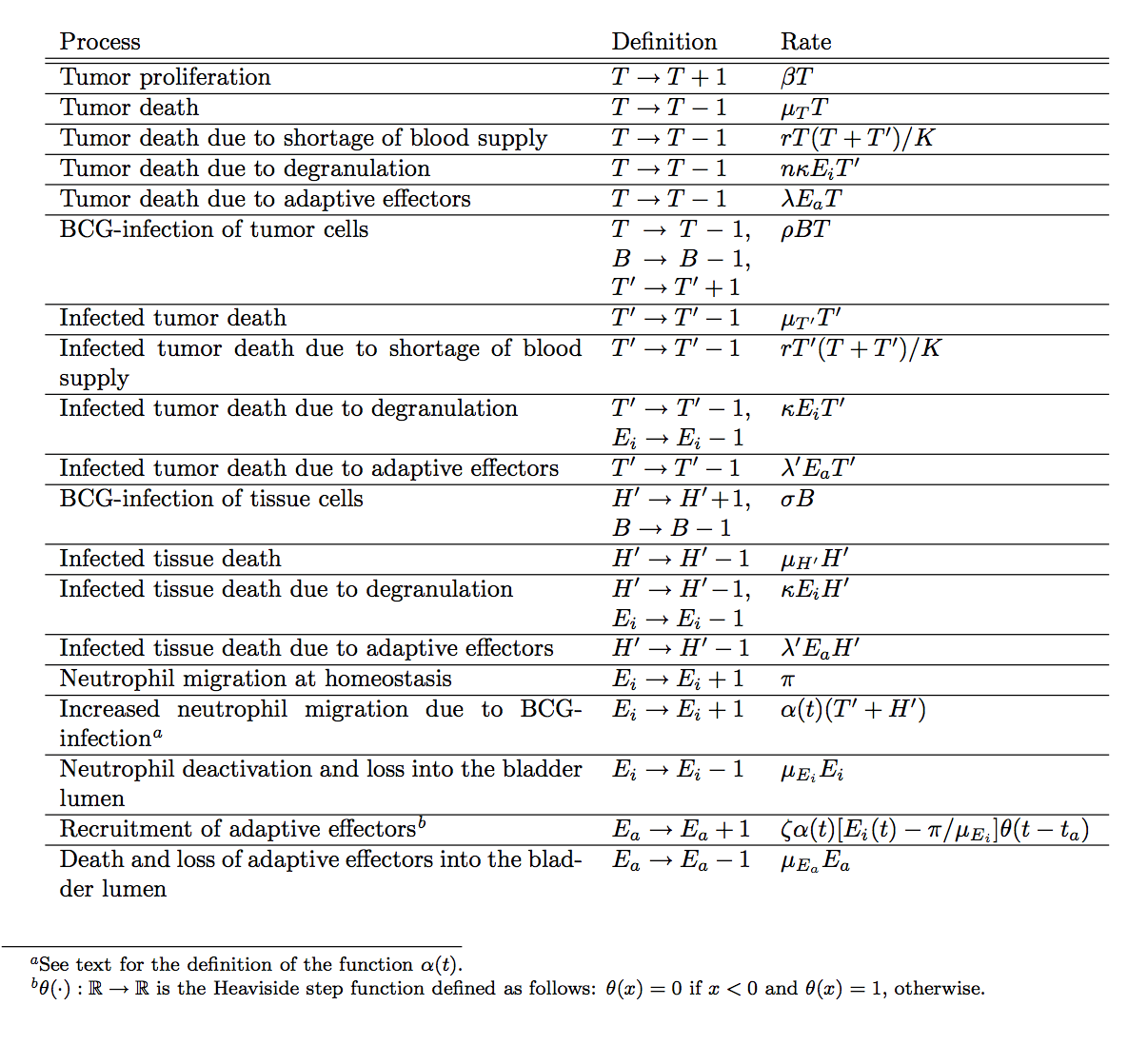

Supplement: Table S2 — Stochastic processes and their corresponding rates. (TIFF) [file pone.0056327.s006.tif]

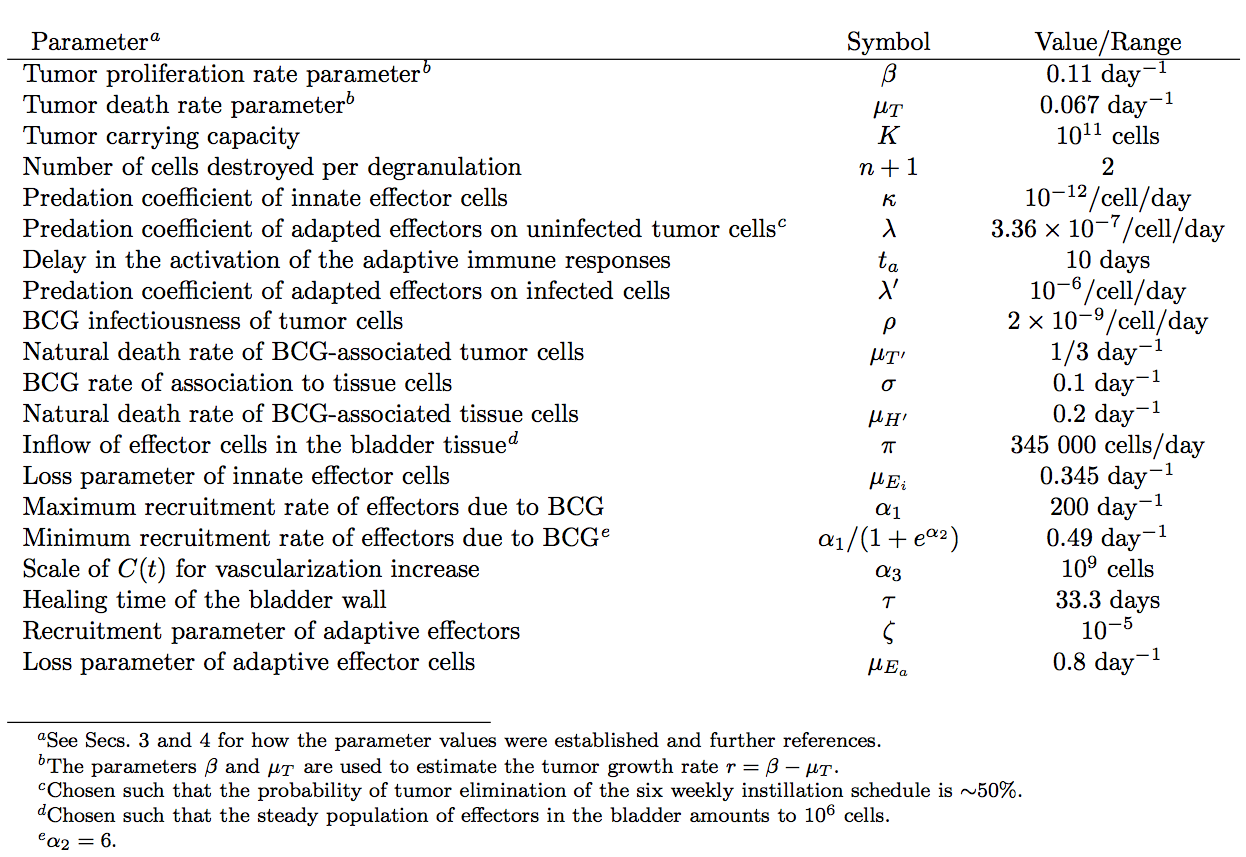

Supplement: Table S3 — Parameters of the model. (TIFF) [file pone.0056327.s007.tiff]
