# Supplementary material for: BCG-Mediated Bladder Cancer Immunotherapy: Identifying Determinants of Treatment Response Using a Calibrated Mathematical Model
Source: PLoS One. 2013 Feb 25;8(2):e56327. doi: 10.1371/journal.pone.0056327 (PMC3581521; doi:10.1371/journal.pone.0056327)
Supplement: Text S1 — Detailed description and numerical analysis of the stochastic mathematical model. (PDF) [file pone.0056327.s008.pdf]

BCG-mediated bladder cancer immunotherapy: Identifying determinants of treatment response using a calibrated mathematical model
Supplemental Material

Cyrill A. Rentsch[1,2], Claire Biot[2–4], Joël R. Gsponer[1,2], Alexander Bachmann[1], Matthew L. Albert[2,4], and Romulus Breban[5]

[1]*Department of Urology, University Hospital of Basel, University of Basel, CH-4031 Basel, Switzerland;*
[2]*Institut Pasteur, Unité d'Immunologie des Cellules Dendritiques, 75015 Paris, France;*
[3]*Mines PARISTECH, 75272 Paris, France;*
[4]*INSERM U818, 75015 Paris, France;*
[5]*Institut Pasteur, Unité d'Epidémiologie des Maladies Emergentes, 75015 Paris, France.*

Contents

1 Model description **2**

2 Stochastic mathematical model **5**

3 Model parameters and simulations **6**

4 Model calibration and parameter uncertainties **9**

1 Model description

We propose a model of interactions between the immune system, bacillus Calmette-Guérin (BCG), bladder and tumor cells during BCG immunotherapy; see the flow diagram depicted in Fig. S1. We extend our previous work [1] where we described the interactions between the *innate* immune system, BCG, bladder and tumor cells during BCG immunotherapy (the diagram of the prior model is included in the gray box in Fig. S1), appending it using a parsimonious description of the adaptive immune system. Our current model has 7 state variables (see Table S1). H denotes the number of healthy cells of the bladder tissue. T denotes the number of tumor cells and B the number of free BCG bacteria in the bladder. We use primed symbols (i.e., H' and T') to denote cell populations that are infected by and/or associated with BCG bacteria. We thus use the symbols H' and T' for the number of BCG-associated tissue and tumor cells, respectively. E_i and E_a denote the number of innate and adaptive effector cells that have extravasated into the bladder, respectively.

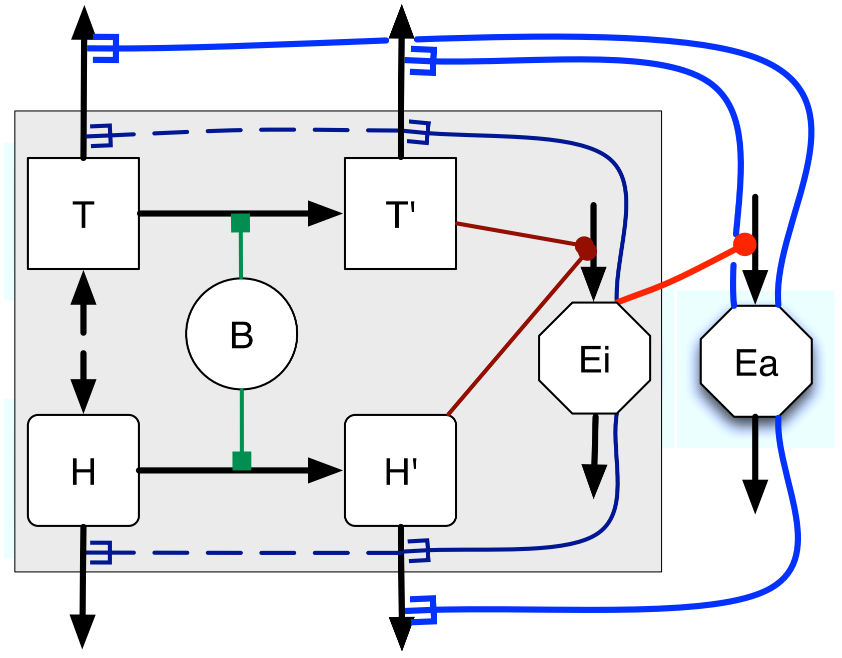

Figure S1: Flow diagram of the model of interactions between the innate immune system and bladder tumor during BCG instillation.

The model makes 13 assumptions. We note that Assumptions 1-5 and 8-13 were used in our previous model describing the interactions between the *innate* immune system, BCG, bladder and tumor cells during BCG immunotherapy [1].

Assumption 1: In absence of therapy, the tumor remains largely undetected by the immune system. The paucity of dendritic cells in the resting bladder mucosa, and the histologic assessment of non-muscle invasive tumors indicate absence of local inflammation. We exclude immune tolerance and/or the presence of regulatory immune cells as one of the mechanism that may hamper BCG-induced tumor immunity, but this may be inferred within other assigned constraints on immune activation.

Assumption 2: During BCG instillations, free BCG associates to tissue and tumor cells (green arrows in Fig. S1). The therapeutic mixture contains both live BCG capable of actively interacting with cells, and dead BCG that is internalized by the cell; hence, we refer to BCG as becoming cell-associated. Cells associated with BCG include cells that have BCG adhered to their plasma membrane, cells that have phagocytosed BCG or components of dead bacilli, cells that have upregulated stress molecules (e.g., MIC-A) as a result of contact

Table S1: State variables of the model along with their biological description. The state variables represent counts of various cell types involved in the interactions between the immune system, tumor cells and BCG.

Variable	Cell type
H	Healthy cells of the bladder tissue
T	Tumor cells in the bladder
B	Free BCG bacteria in the bladder
H'	BCG-associated cells of the bladder tissue
T'	BCG-associated tumor cells
E_i	Innate effector cells in the bladder
E_a	Adaptive effector cells in the bladder

with BCG and cells that have been actively infected by BCG. Experimental data indicates that BCG can become associated with urothelial cells and transitional cell carcinoma cell lines; it has been demonstrated in human studies that small quantities of BCG (∼1% of instilled dose) persist after voiding [2].

Assumption 3: BCG instillations result in an increased recruitment of innate effector cells into the bladder tissue (dark red arrows in Fig. S1) [1].

Assumption 4: Innate effector cells engaging with BCG-associated tissue or tumor cells become activated and degranulate. The degranulation process is the same whether the trigger is a BCG-associated tissue or tumor cell. This assumption is supported by findings of activated innate cells in urine following intravesical therapy and experimental work on co-cultures of BCG or BCG-infected cells and innate immune cells [3].

Assumption 5: Degranulation of an innate effector cell results in the death of the effector cell. Furthermore, tissue or tumor cells in the vicinity of the effector cell may be killed (i.e., see the processes displayed with dark blue arrows in Fig. S1). In the case of neutrophils and inflammatory monocytes, degranulation is known to result in the release of degradative intracellular proteins (e.g., elastase, heparanase, lipases) having the capacity to induce bystander cell death. In addition, activated innate cells may secrete effector cytokines and/or express cell death inducing proteins (e.g., TRAIL). The assumption that NK cells die rapidly after engagement of their effector mechanisms may results in an underestimation of their killing capacity. However, according to clinical data, the relative paucity of NKs recruited to the bladder indicate that even with a high estimate of 10 killing events per NK, their effector function account for <10% of the combined innate response [3].

Assumption 6: Adaptive effector cells are recruited in the bladder as a direct result of the increased number of innate effector cells. However, the initial recruitment is delayed as the adaptive immune system does not react as fast as the innate immune system. For parsimony of the model, we consider a model of signaling by the innate immune system that is based simply on the count of innate effector cells in the bladder. This is meant to embody the activation of migratory dendritic cells that prime adaptive responses, an the local production of chemokines that directly act by attracting lymphocytes into the bladder mucosa. The delay in activation of the adaptive immune system is modeled empirically by simply not allowing adaptive effectors in the bladder sooner than 10 days after the initiation of BCG immunotherapy - the minimal time required to achieve activation of mycobacterial or tumor antigen-specific responses.

Assumption 7: Adaptive effector cells engage equally well with BCG-associated tissue or tumor cells. Adaptive effector cell also engage, but less effectively, with BCG-unassociated tumor cells. As a result of the engagement process, only the target cell is destroyed. For parsimony of the model, we consider only one population of adapted effector cells that, on average, fulfills the roles of a heterogeneous mix of lymphocytes that occurs in reality. This assumption reduces considerably the number of parameters needed to describe the adaptive immune system and makes possible the calibration of the model to clinical data. By offsetting the potency of engagement of BCG-associated vs. unassociated cells, we may introduce the notion of affinity for antigen, which is expected to be greater for microbial antigen as compared to tumor antigen [4].

The model runs as follows. Before immunotherapy, only three of seven cell populations are present: H, T

and E_i. The interactions between these cell populations are negligible and their corresponding compartments are disconnected. The processes that take place for each of these independent compartments are "birth" (i.e., cell migration and/or local proliferation) and death. During BCG instillations, three new populations of cells emerge creating dynamic interactions between all the compartments. B associates to tissue and tumor cells (green arrows in Fig. S1), inducing transitions of cells from H to H' and from T to T' (horizontal black arrows in Fig. S1). The presence of H' and T' cells triggers increased migration of effector cells into the bladder tissue. H' and T' cells activate innate immune effectors E_i. In turn, E_i cells target H' and T' destroying them and neighboring H and T cells by degranulation. Furthermore, the excess number of E_i cells triggers recruitment of adaptive effector cells E_a with a much enhanced killing capacity. Finally, the production of inflammation induced chemattractants, the activation and the exfoliation of bladder endothelium results in an ensemble of phenomena that increase the effector cell migration to the bladder. It is known that effector cell migration increases from the first to the third BCG instillation after which time it starts to plateau [3].

Table S2: Stochastic processes and their corresponding rates.

Process	Definition	Rate
Tumor proliferation	$T \to T+1$	βT
Tumor death	$T \to T-1$	$\mu_T T$
Tumor death due to shortage of blood supply	$T \to T-1$	$rT(T+T')/K$
Tumor death due to degranulation	$T \to T-1$	$n\kappa E_i T'$
Tumor death due to adaptive effectors	$T \to T-1$	$\lambda E_a T$
BCG-infection of tumor cells	$T \to T-1$, $B \to B-1$, $T' \to T'+1$	ρBT
Infected tumor death	$T' \to T'-1$	$\mu_{T'} T'$
Infected tumor death due to shortage of blood supply	$T' \to T'-1$	$rT'(T+T')/K$
Infected tumor death due to degranulation	$T' \to T'-1$, $E_i \to E_i-1$	$\kappa E_i T'$
Infected tumor death due to adaptive effectors	$T' \to T'-1$	$\lambda' E_a T'$
BCG-infection of tissue cells	$H' \to H'+1$, $B \to B-1$	σB
Infected tissue death	$H' \to H'-1$	$\mu_{H'} H'$
Infected tissue death due to degranulation	$H' \to H'-1$, $E_i \to E_i-1$	$\kappa E_i H'$
Infected tissue death due to adaptive effectors	$H' \to H'-1$	$\lambda' E_a H'$
Neutrophil migration at homeostasis	$E_i \to E_i+1$	π
Increased neutrophil migration due to BCG-infection[a]	$E_i \to E_i+1$	$\alpha(t)(T'+H')$
Neutrophil deactivation and loss into the bladder lumen	$E_i \to E_i-1$	$\mu_{E_i} E_i$
Recruitment of adaptive effectors[b]	$E_a \to E_a+1$	$\zeta\alpha(t)[E_i(t)-\pi/\mu_{E_i}]\theta(t-t_a)$
Death and loss of adaptive effectors into the bladder lumen	$E_a \to E_a-1$	$\mu_{E_a} E_a$

[a] See text for the definition of the function $\alpha(t)$.
[b] $\theta(\cdot) : \mathbb{R} \to \mathbb{R}$ is the Heaviside step function defined as follows: $\theta(x) = 0$ if $x < 0$ and $\theta(x) = 1$, otherwise.

2 Stochastic mathematical model

Since we are particularly interested in the phenomenon of tumor elimination, we develop a stochastic mathematical model (i.e., continuous-time Markov chain) where the cell counts are described by integers and thus can go extinct. We make further assumptions to express the rates of the stochastic model of BCG immunotherapy in a parsimonious way.

Table S3: Parameters of the model.

Parameter[a]	Symbol	Value/Range
Tumor proliferation rate parameter[b]	β	0.11 day^{-1}
Tumor death rate parameter[b]	μ_T	0.067 day^{-1}
Tumor carrying capacity	K	10^{11} cells
Number of cells destroyed per degranulation	$n+1$	2
Predation coefficient of innate effector cells	κ	10^{-12}/cell/day
Predation coefficient of adapted effectors on uninfected tumor cells[c]	λ	3.36×10^{-7}/cell/day
Delay in the activation of the adaptive immune responses	t_a	10 days
Predation coefficient of adapted effectors on infected cells	λ'	10^{-6}/cell/day
BCG infectiousness of tumor cells	ρ	2×10^{-9}/cell/day
Natural death rate of BCG-associated tumor cells	$\mu_{T'}$	1/3 day^{-1}
BCG rate of association to tissue cells	σ	0.1 day^{-1}
Natural death rate of BCG-associated tissue cells	$\mu_{H'}$	0.2 day^{-1}
Inflow of effector cells in the bladder tissue[d]	π	345 000 cells/day
Loss parameter of innate effector cells	μ_{E_i}	0.345 day^{-1}
Maximum recruitment rate of effectors due to BCG	α_1	200 day^{-1}
Minimum recruitment rate of effectors due to BCG[e]	$\alpha_1/(1+e^{\alpha_2})$	0.49 day^{-1}
Scale of $C(t)$ for vascularization increase	α_3	10^9 cells
Healing time of the bladder wall	τ	33.3 days
Recruitment parameter of adaptive effectors	ζ	10^{-5}
Loss parameter of adaptive effector cells	μ_{E_a}	0.8 day^{-1}

[a] See Secs. 3 and 4 for how the parameter values were established and further references.
[b] The parameters β and μ_T are used to estimate the tumor growth rate $r = \beta - \mu_T$.
[c] Chosen such that the probability of tumor elimination of the six weekly instillation schedule is ∼50%.
[d] Chosen such that the steady population of effectors in the bladder amounts to 10^6 cells.
[e] $\alpha_2 = 6$.

Assumption 8: We assume that tumor undergoes logistic growth. Modeling the population dynamics of BCG-free tumor cells, we assume that the tumor grows into the bladder lumen and does not impinge upon surrounding tissue. As such, its size is limited only by the blood supply. We choose the logistic model (i.e., the Verhulst model) to describe the dynamics of the tumor cell population in absence of BCG therapy [5,6]. Notably, our model will be applied to the description of post-resection BCG immunotherapy where the number of tumor cells is significantly less than the carrying capacity (i.e., the maximum number of tumor cells that the blood supply can sustain). Consequently, the logistic model will be used in the regime of exponential growth. Therefore, the results presented in this paper are not restricted by our choice of the logistic model and apply to all models of tumor cell replication in their regime of exponential growth.

Assumption 9: We do not use explicit equations to model the dynamics of the healthy tissue cell population. Rather, we consider that this population is very large when compared with the other cell populations of interest. This assumption is based on clinical information that BCG therapy does not result in the perforation of the bladder wall nor frank disruption of bladder function.

Assumption 10: We use mass-action to describe the mixing between the cell populations. This is supported by BCG dispersion during intravesical therapy, adequate vascularization of all aspects of the bladder wall and non-specific migration patterns of innate immune cells as they enter inflamed tissue.

Assumption 11: The number of BCG bacteria that die during instillation (i.e., 2 hours) is negligible. The kinetics of the natural death of BCG is slow and the host response is minimal within the first hours of BCG instillation. Thus, for modeling purposes, we neglect BCG death during the course of instillation therapy.

Assumption 12: BCG-associated cells do not undergo local proliferation. This is supported by experimental data suggesting that BCG negatively impacts the cell growth of bladder tumor cell lines [7].

Assumption 13: The migration of innate effector cells increases due to vascularization of the bladder wall. This increase is proportional to the number of BCG-associated cells present in the bladder. Furthermore, the cumulative number of cells destroyed by degranulation stimulates neo-vascularization in a sigmoidal fashion. This is counteracted by homeostatic pressure (i.e., healing of the bladder wall) which is modeled as a discount factor of the cumulative number of cells destroyed by degranulation. This assumption is supported by the observation that migration of effector cells is linked to the vascularization of the bladder wall, which evolves as a function of inflammation induced cell death [3, 8]. As such, the increase in the inflow of immune cells depends on the number of BCG-associated cells present in the bladder, which are triggers for activation and effector activity of innate immune cells.

We now define transition rates for each of the processes depicted in the flow diagram (Fig. S1); see Table S2. The parameters of the model are explained in Table S3. The processes of vascularization and healing are modeled by a sigmoidal function $\alpha(t)$ given by

$$\alpha(t) = \frac{\alpha_1}{1 + \exp[\alpha_2 - C(t)/\alpha_3]}, \tag{2.1}$$

where $C(t)$ represents the number of destroyed cells discounted by a negative exponential "healing" factor

$$C(t) = \int_0^t du(n+1)\{\kappa E_i(u)[T'(u) + H'(u)] + \lambda E_a(u)T(u) + \lambda' E_a(u)[T'(u) + H'(u)]\}e^{-u/\tau}. \tag{2.2}$$

This completes the definition of our continuous-time Markov chain model. Various algorithms may be used for the numerical integration of this stochastic model. We used the so-called *efficient tau-leaping method* [9] to speed up the computations.

3 Model parameters and simulations

We carried out simulations in order to establish values for the parameters of our model. First, we investigated tumor growth in absence of BCG therapy. Figure S2 shows a simulation with initial conditions: $T(0) = 1$, $T_i(0) = 0$, $H_i(0) = 0$, $E_i(0) = 10^6$ and $E_a(0) = 0$. The simulation includes resection of tumor from the carrying capacity of $\sim 10^{11}$ cells [10] to $\sim 10^6$ cells (e.g., in case of carcinoma in situ). The proliferation β, death μ_T and growth $r = \beta - \mu_T$ rate parameters are tuned according to our previous work [1] such that the re-growth of the tumor to a visible size (i.e., $\sim 10^8$ cells) takes about three months [11].

Figure S3 shows a simulation of the dynamics of cell populations during BCG therapy. The initial conditions are: $T(0) = 10^6$, $T_i(0) = 0$, $H_i(0) = 0$, $E_i(0) = 10^6$ and $E_a(0) = 0$. The BCG amount was chosen as follows. Commercial vials of lyophilized BCG used for immunotherapy contain $\sim 10^8$ live bacteria (or colony forming units – CFU), representing 5-10% of the total number of bacteria in the preparation [12]. During BCG instillations, live BCG is capable of actively interacting with cells while dead BCG may be internalized by cells. To take into account all these interactions, we assumed that, for each instillation, B starts at 10^9 bacteria (of which only 10^8 is considered live BCG) but then it is set to zero at the end of the instillation. The parameters ρ and σ quantifying the interactions between BCG and tumor and tissue cells, respectively, were chosen such that, at the end of an instillation: (i) $\sim 1\%$ of the tumor cells are BCG associated [1], and (ii) $\sim 99\%$ of the free BCG is flushed [2, 13]. Panel **A** of Fig. S3 shows the percent of free BCG that is present in the bladder during the instillations. Note that $\sim 99\%$ of the free BCG is flushed after each instillation, according to data.

The parameters of innate effector cells are chosen as follows. Innate effector cells are lost primarily due to deactivation, death and fallout into urine. Kinetics data show that the expected lifetime of innate effectors is ~ 8 hours [3, 14]; hence, we arrived at the μ_{E_i} estimate given in Table S3. Based on the typical density of innate effectors in the blood (see Ref. [15], Table 22-1), we estimated that, in absence of immunotherapy,

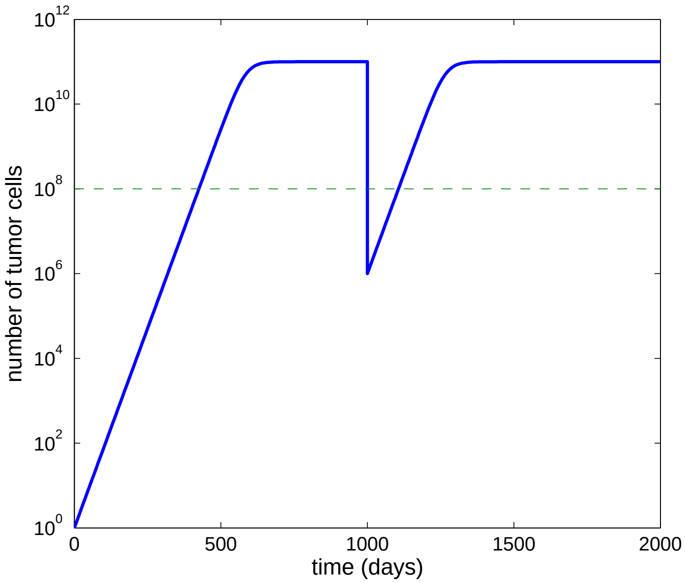

Figure S2: Simulation of logistic tumor growth, resection and tumor re-growth. The horizontal dashed line represents the approximate size of the tumor when the tumor is visible on the bladder wall.

the steady population of innate effectors in the bladder amounts to $\sim 10^6$ cells. Combining this with μ_{E_i}, we obtained the inflow of effector cells in the bladder tissue in the range of $\sim$ 345 000 cells/day. See also Ref. [1] for these parameter values. The expected number of cells destroyed per degranulation $(n+1)$ was also obtained from Ref. [1].

Data on the prime/boost effect provides two major references to determine parameter values. First, the prime/boost effect occurs at the third BCG instillation [3]. Second, data provides E_a and E_i counts *before and after* the prime/boost effect [3]. Imposing these conditions on the output of our model, we obtained the parameters $\alpha_{1,2,3}$, the E_a recruitment parameter ζ, and the predation coefficient of innate effector cells κ.

The healing time of the bladder wall τ was determined based on the fact that most lesions inside the bladder are completely restored at cystoscopy three months after transurethral resection of the bladder wall. Hence, the sigmoid $\alpha(t)$ describing vascularization and healing of the bladder wall was fully parameterized. Together with the value of t_a [3], the delay in the activation of the adaptive immune responses, $\alpha(t)$ was used to modulate recruitment of E_a and E_i.

It is also important to note that the number of tumor cells left after resection is small. Hence, it is natural to assume that the prime/boost effect would occur essentially the same whether or not there are tumor cells left in the bladder; i.e., whether $T(0) = 0$ or $\sim 10^6$ cells. This constraint was used to determine the predation coefficient of adapted effectors on BCG-associated cells, λ'.

Simulation results on the modeled prime/boost effect are reported in Fig. S3. The dynamics of the tumor cells (panel **B**) is marked by slight increase due to local proliferation before the prime/boost of the immune response followed by steep decrease due to the next BCG instillations. Panels **C** and **D** represent the dynamics of the population of BCG-associated tumor cells and BCG-associated tissue cells, respectively. The population of BCG-associated tumor cells is fairly small and undergoes extinction in between the BCG instillations. Panel **E** shows the dynamics of the population of innate effector cells. Note the significant growth in the number of effector cells after the third instillation, corresponding to the prime/boost effect. Panel **F** shows the dynamics of the population of effector cells of the adapted immune system that is also modulated by the prime/boost effect. Furthermore, the effector cells of the adapted immune system arrive late in the bladder compared to those of the innate immune system.

The parameters describing the natural death rate of BCG-associated tumor cells $\mu_{T'}$ and that of BCG-

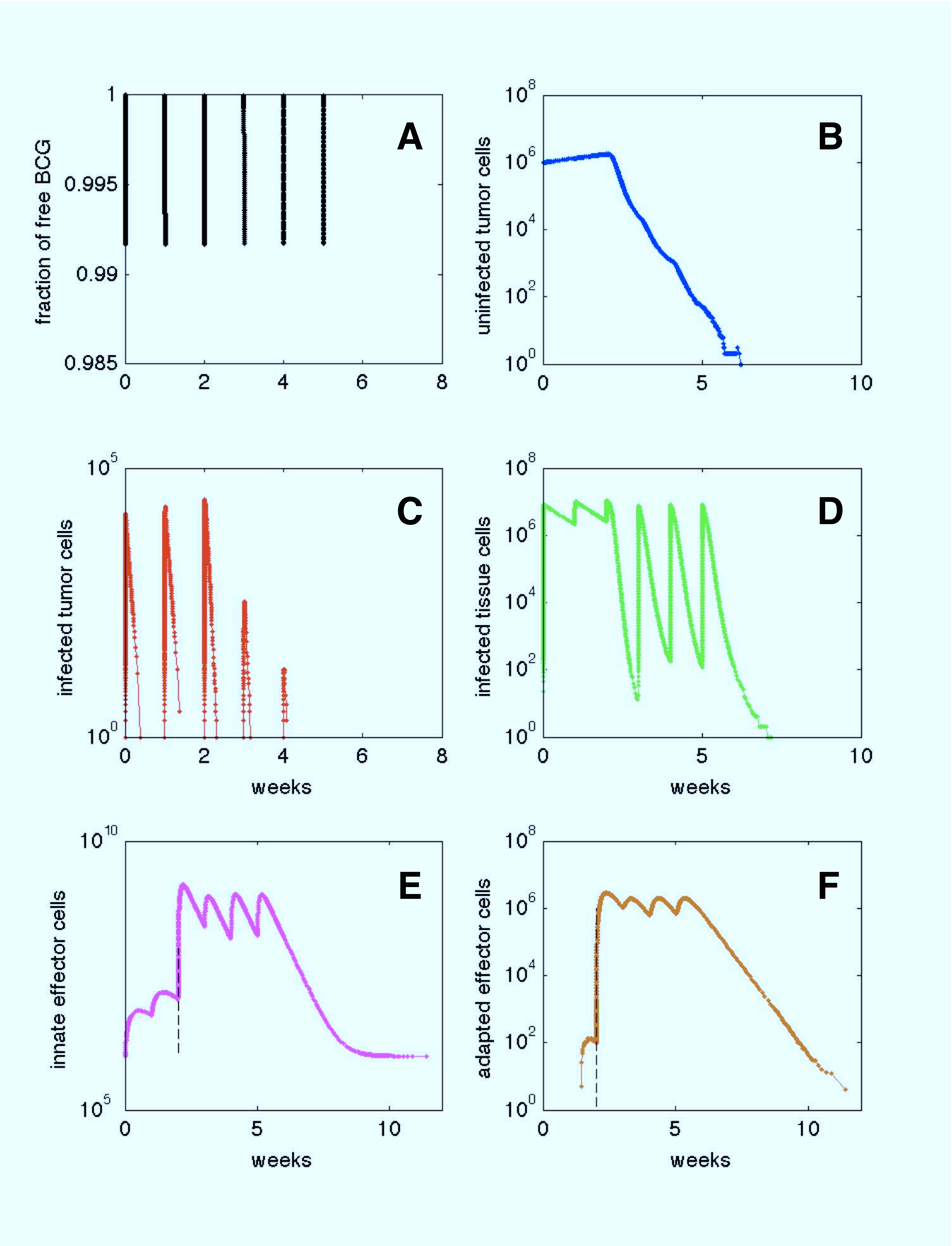

Figure S3: Simulation of population dynamics of cells during and after a six-week course of intravesical BCG therapy: BCG (panel **A**), tumor cells (panel **B**), BCG-associated tumor cells (panel **C**), BCG-associated tissue cells (panel **D**), innate effector cells (panel **E**), adaptive effector cells (panel **F**). Note the modeling of the prime/boost response of the innate and adapted immune systems which occurs after the third instillation (in particular, note panels **E** and **F**).

associated tissue cells $\mu_{H'}$ were taken from Ref. [1] and are in agreement with data [16]. The parameter describing the loss of adaptive effector cells μ_{E_a} was considered to be 0.8 day^{-1} [3]. However, this parameter

value is difficult to select given the diversity of adapted effector cells that we gathered in the E_a population. Hence, in Sec. 4 we discuss sensitivity analyses of our results versus this parameter. Lastly, the average predation coefficient of adapted effectors on uninfected tumor cells λ was obtained by calibration of our model to clinical data; see Sec. 4.

4 Model calibration and parameter uncertainties

The key parameter for tumor elimination is the predation coefficient of adapted effectors on uninfected tumor cells, λ. It is calibrated such that the probability of tumor elimination of the six weekly-instillation schedule is $\sim 50\%$ where the initial conditions (at time two weeks post-surgical resection, taken as the minimum time delay for initiation of BCG therapy), were chosen as follows $T(0) = 10^6$, $T_i(0) = 0$, $H_i(0) = 0$, $E_i(0) = 10^6$ and $E_a = 0$.

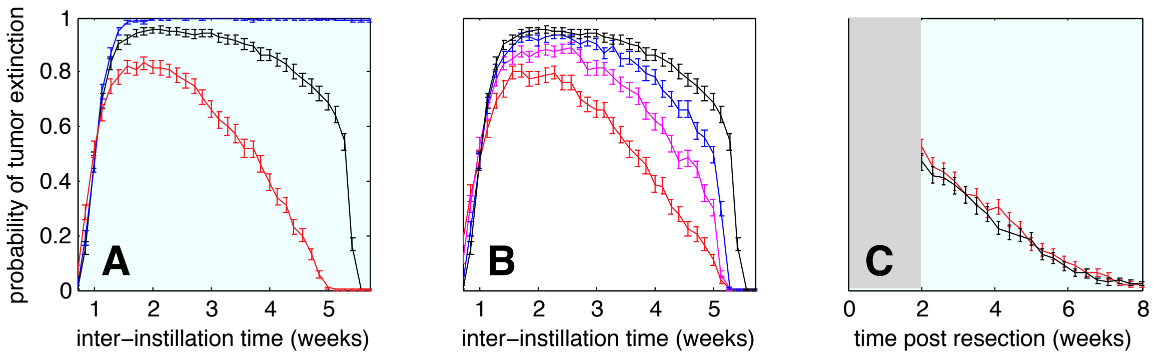

Figure S4: Sensitivity analyses for Figs. 2A and 3 of the main text. **A**. Sensitivity analysis of Fig. 3 with changing the rate of loss of adapted effectors, μ_{E_a}. The blue, black (also shown in Fig. 3) and red curves correspond to $\mu_{E_a} = 0.4$, 0.8 and 1.6 day^{-1}, respectively. For every parameter set, the model has been re-calibrated such that six weekly instillation of BCG therapy yield $\sim 50\%$ chance of cure. **B**. Sensitivity analysis of Fig. 3 with varying the number of tumor cells found in the bladder before initiating BCG therapy, $T(0)$. The black (also shown in Fig. 3), blue, magenta and red curves correspond to $T(0) = 10^6$, 10^5, 10^4 and 10^3 tumor cells. The model has been re-calibrated for each value of $T(0)$, accordingly. These numerics suggest that our prediction that a two-week inter-instillation interval would significantly improve therapeutic outcome is robust. **C**. Sensitivity analysis of Fig. 2A with changing the number of tumor cells before initiating BCG therapy, $T(0)$. The black (also shown in Fig. 2A) and red curves correspond to $T(0) = 10^6$ and 10^3 tumor cells.

We performed calibration of the model for other sets of parameters than that given in Table S3. For example, changing the loss parameter μ_{E_a} from 0.8 day^{-1} to 1.6 day^{-1} and 0.4 day^{-1}, we obtained λ approximately equal to 5.52×10^{-7}/cell/day and 2.17×10^{-7}/cell/day, respectively. We used these parameter sets to run further simulations of BCG regimens with varying the inter-instillation interval. The results are presented in Fig. S4**A**. We found that the success of BCG immunotherapy is sensitive to the rate of loss of effectors of the adaptive immune system from the bladder. However, our prediction that a two-week inter-instillation interval would significantly improve therapeutic outcome (as compared to a one-week inter-instillation interval) remains valid with changes in the loss rate.

We performed calibration for other sets of initial conditions that differ in $T(0)$, the number of tumor cells found in the bladder before BCG therapy, while keeping all the other parameters as given by Table S3. For $T(0)$ equals 10^3, 10^4 and 10^5 cells, we obtained λ approximately equal to 1.80×10^{-7}/cell/day, 2.35×10^{-7}/cell/day and 2.86×10^{-7}/cell/day, respectively. We then ran simulations of BCG regimens with varying the inter-instillation interval (Fig. S4**B**) for these different sets of initial conditions. Simulation results show similar patterns in all these scenarios. Although the probability of cure is sensitive to the

number of tumor cells before immunotherapy, the prediction that a two-week inter-instillation interval would significantly improve therapeutic outcome is robust.

We further used the above parameter sets to investigate how the probability of tumor extinction changes with the duration of time between resection and initiation of therapy (Fig. S4**C**). In this case, we found that therapeutic success as a function of the time interval between resection and BCG immunotherapy is not very sensitive with changing the number of tumor cells left in the bladder after resection.

References

[1] Breban R, Bisiaux A, Biot C, Rentsch C, Bousso P, et al. (2012) Mathematical model of tumor immunotherapy for bladder carcinoma identifies the limitations of the innate immune response. OncoImmunology 1: 9-17.

[2] Durek C, Richter E, Basteck A, Rüsch-Gerdes S, Gerdes J, et al. (2001) The fate of bacillus Calmette-Guérin after intravesical instillation. J Urol 165: 1765-8.

[3] Bisiaux A, Thiounn N, Timsit M, Eladaoui A, Chang H, et al. (2009) Molecular analyte profiling of the early events and tissue conditioning following intravesical bacillus Calmette-Guérin therapy in patients with superficial bladder cancer. J Urol 181: 1571–80.

[4] de Visser K, Schumacher T, Kruisbeek A (2003) CD8(+) T cell tolerance and cancer immunotherapy. J Immunother 26: 1–11.

[5] Aroesty J, Lincoln T, Shapiro N, Boccia G (1973) Tumor growth and chemotherapy: Mathematical methods, computer simulations, and experimental foundations. Mathematical Biosciences 17: 243 - 300.

[6] Bunimovich-Mendrazitsky S, Shochat E, Stone L (2007) Mathematical model of BCG immunotherapy in superficial bladder cancer. Bull Math Biol 69: 1847-70.

[7] Jackson A, Alexandroff A, Fleming D, Prescott S, Chisholm G, et al. (1994) Bacillus Calmette-Guérin (BCG) organisms directly alter the growth of bladder-tumor cells. International Journal of Oncology 5: 697–703.

[8] Saban MR, Backer JM, Backer MV, Maier J, Fowler B, et al. (2008) VEGF receptors and neuropilins are expressed in the urothelial and neuronal cells in normal mouse urinary bladder and are upregulated in inflammation. Am J Physiol Renal Physiol 295: F60-72.

[9] Cao Y, Gillespie DT, Petzold LR (2006) Efficient step size selection for the tau-leaping simulation method. J Chem Phys 124: 044109.

[10] Sylvester RJ, van der Meijden APM, Oosterlinck W, Witjes JA, Bouffioux C, et al. (2006) Predicting recurrence and progression in individual patients with stage Ta T1 bladder cancer using EORTC risk tables: a combined analysis of 2596 patients from seven EORTC trials. Eur Urol 49: 466-5; discussion 475-7.

[11] Thomas F, Noon AP, Rubin N, Goepel JR, Catto JWF (2012) Comparative outcomes of primary, recurrent, and progressive high-risk non-muscle-invasive bladder cancer. Eur Urol .

[12] Behr M (2002) BCG–different strains, different vaccines? The Lancet Infectious Diseases 2: 86–92.

[13] Siatelis A, Houhoula DP, Papaparaskevas J, Delakas D, Tsakris A (2011) Detection of bacillus Calmette-Guérin (mycobacterium bovis BCG) DNA in urine and blood specimens after intravesical immunotherapy for bladder carcinoma. J Clin Microbiol 49: 1206-8.

[14] Dancey JT, Deubelbeiss KA, Harker LA, Finch CA (1976) Neutrophil kinetics in man. J Clin Invest 58: 705-15.

[15] Alberts B, Wilson JH, Hunt T (2008) Molecular biology of the cell. New York: Garland Science, 5th ed.

[16] Secanella-Fandos S, Luquin M, Esther Julián (2012) Connaught and Russian showed the highest direct antitumoral effects among different BCG substrains. J Urol doi: 10.1016/j.juro.2012.09.049.
